# Organic cation transporter 1 (OCT1) is involved in pentamidine transport at the human and mouse blood-brain barrier (BBB)

**DOI:** 10.1371/journal.pone.0173474

**Published:** 2017-03-31

**Authors:** Gayathri N. Sekhar, Ana R. Georgian, Lisa Sanderson, Gema Vizcay-Barrena, Rachel C. Brown, Paula Muresan, Roland A. Fleck, Sarah A. Thomas

**Affiliations:** 1 King’s College London, Institute of Pharmaceutical Science, Waterloo, London United Kingdom; 2 King’s College London, Centre for Ultrastructural Imaging, King’s College London, London Bridge United Kingdom; University of Cambridge, UNITED KINGDOM

## Abstract

Pentamidine is an effective trypanocidal drug used against stage 1 Human African Trypanosomiasis (HAT). At the blood-brain barrier (BBB), it accumulates inside the endothelial cells but has limited entry into the brain. This study examined transporters involved in pentamidine transport at the human and mouse BBB using hCMEC/D3 and bEnd.3 cell lines, respectively. Results revealed that both cell lines expressed the organic cation transporters (OCT1, OCT2 and OCT3), however, P-gp was only expressed in hCMEC/D3 cells. Polarised expression of OCT1 was also observed. Functional assays found that ATP depletion significantly increased [^3^H]pentamidine accumulation in hCMEC/D3 cells (***p<0.001) but not in bEnd.3 cells. Incubation with unlabelled pentamidine significantly decreased accumulation in hCMEC/D3 and bEnd.3 cells after 120 minutes (***p<0.001). Treating both cell lines with haloperidol and amantadine also decreased [^3^H]pentamidine accumulation significantly (***p<0.001 and **p<0.01 respectively). However, prazosin treatment decreased [^3^H]pentamidine accumulation only in hCMEC/D3 cells (*p<0.05), and not bEnd.3 cells. Furthermore, the presence of OCTN, MATE, PMAT, ENT or CNT inhibitors/substrates had no significant effect on the accumulation of [^3^H]pentamidine in both cell lines. From the data, we conclude that pentamidine interacts with multiple transporters, is taken into brain endothelial cells by OCT1 transporter and is extruded into the blood by ATP-dependent mechanisms. These interactions along with the predominant presence of OCT1 in the luminal membrane of the BBB contribute to the limited entry of pentamidine into the brain. This information is of key importance to the development of pentamidine based combination therapies which could be used to treat CNS stage HAT by improving CNS delivery, efficacy against trypanosomes and safety profile of pentamidine.

## Introduction

Pentamidine is an aromatic diamidine that is used clinically to treat the haemolymphatic stage of Human African Trypanosomiasis (HAT). It is also used to treat American cutaneous leishmaniasis and as a prophylactic for *Pneumocystis jirovecii pneumonia* [1]. HAT is a major public health problem in sub-Saharan Africa and is classified as one of the most neglected tropical diseases. It is often fatal without chemotherapy. It is a parasitic disease caused by a tsetse fly transmitted eukaryotic parasite called Trypanosoma brucei (T.b.). There are two sub-types of this parasite, *T*.*b*. *gambiense* and *T*.*b*. *rhodesiense*. The parasites enter the vascular system early in the course of the infection (the haemolymphatic stage or stage 1 of HAT) and then gradually enter the central nervous system (CNS) (the meningoencephalitic stage or stage 2 of HAT). Pentamidine is effective against *T*.*b*. *gambiense* before the parasite spreads into the CNS, but is ineffective in late stage 2 due to its limited ability to cross the blood-brain barrier (BBB) [2].

Pentamidine entry into the parasite and the host via membrane transporters has been suggested to be key in its mode of action. Pentamidine is a dicationic molecule at physiological pH, and is water soluble (octanol-saline partition coefficient of 0.14368 ± 0.00337 [2]. Consequently it has a low permeability to cross biological membranes by passive diffusion. Therefore, the drug must enter trypanosomes through facilitated diffusion using a selective transporter. Pentamidine accumulation within the trypanosome was found to involve multiple transporters including an adenosine-sensitive pentamidine transporter (P2), an adenosine-insensitive high affinity pentamidine transporter 1 (HAPT1, also called aquaglyceroporin 2 (AQP2)) and an adenosine-insensitive low affinity pentamidine transporter 1 (LAPT1), with K_m_ values of 0.26 μM, 36 nM and 56 μM respectively [3–6]. Interestingly, loss of P2 function in trypanosomes causes drug resistance against pentamidine [7,8]. Further research found that the P2 transporter transports melarsoprol (a stage 2 HAT drug) with higher affinity than pentamidine, and HAPT1 transports pentamidine with a higher affinity than melarsoprol. This transporter specificity also explains the cross-resistance commonly observed between pentamidine and melarsoprol; parasites that were resistant to pentamidine and melarsoprol were all found to have mutations or deletions of AQP2 as well as P2 [5,9].

Such observations help elucidate the mechanisms of pentamidine pharmacokinetics in humans. For example, they suggest that pentamidine would require transporters to efficiently cross the brain capillary endothelial cells and reach brain tissue. Indeed, Sanderson et al. (2009) [2] observed that pentamidine is subjected to efflux by ATP-binding cassette (ABC) transporters present at the mouse BBB. When P-gp (mdr1a/mdr1b targeted mutation) knockout mice were compared to wild-type control (FVB) mice, there was significantly increased (two-fold) accumulation of pentamidine into the brain. Pentamidine was also found to accumulate more in the endothelial cell fractions of the brain than the brain parenchyma homogenate. This suggests that a transporter exists for pentamidine at the luminal membrane of the human brain endothelial cells that transports the drug into the cell before it is effluxed back into the blood. Overall this 2009 study implicated multiple transporters at the BBB for pentamidine. This present study builds on the knowledge obtained from wild type and transgenic mice *in vivo* and focuses on identifying these transporter(s) at the human and mouse BBB by using sensitive *in vitro* methods

A previous study conducted on human organic cationic transporter (hOCT)-expressing Chinese hamster ovary cells showed that pentamidine is a substrate for hOCT1 (K_m_ value of 36.4 μM, [10]. OCTs belong to the SLC22 family of transporters and are polyspecific for cationic organic molecules, including several therapeutic drugs. Three subtypes of OCTs have been functionally identified at the BBB—OCT1, 2,and 3 [11–14]. Importantly there are other transporters for organic cations expressed at the BBB including organic cation transporters novel (OCTN), multi-drug and toxin extrusion transporters (MATE), plasma membrane monoamine transporter (PMAT), concentrated nucleoside transporters (CNTs) and equilibrative nucleoside transporters (ENT). To date there have been no studies investigating the role of OCTs, OCTN, MATE or PMAT in the transport of pentamidine at the human or mouse BBB.

The hypothesis for the present study is that pentamidine enters the BBB by transporters of organic cations expressed at the luminal membrane and is then extruded by ABC transporters also present at the luminal membrane. This is explored in this study using *in vitro* models of the mouse and human BBB and targeted transporter inhibition studies. This study also examined transporter protein expression and localisation using Western blot analysis, confocal microscopy, and Transmission Electron Microscopy (TEM).

This information is invaluable to those developing new diamidine compounds plus those who are interested in reformulating pentamidine to allow improved CNS access, safer treatment and efficacy against HAT [15]. It may also be of interest to those interested in American cutaneous leishmaniasis as pentamidine transporters are expressed in certain Leishmania species [16–18].

## Materials and methods

### Materials

[^3^H(G)]pentamidine (mol. wt., 340.4; specific activity, 31.9 Ci/mmol; 99% radiochemical purity) was custom synthesised and tritiated (Moravek Biochemicals, Brea, CA). [^14^C(U)]sucrose (536 mCi/mmol, 99.4% purity) was purchased from Moravek Biochemicals. Pentamidine (1,5-bis-4′-amidinophenoxypentane) isethionate salt, haloperidol, amantadine hydrochloride, dexamethasone, ko143 hydrate, corticosterone, prazosin hydrochloride, adenosine, 2-deoxy-D-glucose (2-DG), dimethyl sulfoxide (DMSO), Dulbecco’s modified eagle’s medium (DMEM), HEPES (1 M), 10x non-essential amino acids, foetal bovine serum (FBS), Immobilon(R)-PSQ Polyvinylidene difluoride (PVDF) membranes were purchased from Sigma-Aldrich Company Ltd (Poole, Dorset, UK). MK571 and fludarabine were purchased from Cambridge Bioscience, Cambridge, UK. Pheophorbide A and N-methylnicotinamide were purchased from Santa Cruz Biotechnology, Germany. ko143 was purchased from Tocris Bioscience, Bristol, UK. The EGM-2MV BulletKit was purchased from Lonza (Basel, Switzerland). All cultureware was Nunclon brand and purchased from Thermo Scientific, UK. Rat tail collagen 1, penicillin-streptomycin, trypsin-EDTA (10x) were purchased from Gibco, Life Technologies (Paisley, UK). Anti-MDR1/P-gp primary antibody [EPR10364-57] (ab170904), anti-BCRP/ABCG2 antibody (ab3380), anti-MRP4 antibody [M4I-10] (ab15602), anti-SLC22A3 (OCT1) antibody (ab55916), anti-SLC22A2 (OCT2) antibody [EPR11248] (ab170871, anti-SLC22A3 (OCT3) antibody (ab183071), goat anti-Rabbit IgG H&L (HRP) (ab6721), rabbit Anti-Rat IgG H&L (HRP) (ab6734), goat Anti-Rabbit IgG H&L (Alexa Fluor^®^ 488) (ab181448), anti-GAPDH antibody (ab9485) were purchased from Abcam, Cambridge, UK. Anti-BCRP/ABCG2 antibody (4477S) was purchased from New England Biolabs (Hertfordshire, UK). Goat anti-mouse IgG2a cross-adsorbed secondary antibody, (Alexa Fluor 546) (Cat A-21133) was a gift fromDr Ana Georgian. Anti-alpha tubulin antibody, clone EP1332Y was purchased from Millipore (UK) Limited, Watford, UK.**)**. HepG2 cell lysate was purchased from Enzo Life Sciences, Exeter, UK. Proteoblock™ protease inhibitor cocktail, and Lane Marker Reducing Sample Buffer were purchased from Thermo Fisher Scientific Biosciences GmbH, Germany. Precision Plus Protein™ Dual Color Standards protein ladder was purchased from Bio-Rad Laboratories, Hemel Hempstead, UK.

### Cell culture

The human cerebral microvessel endothelial cell/D3 (hCMEC/D3) is the most promising immortalized human BBB cell line available today, exhibiting many of the characteristics that are essential for a good predictive BBB *in vitro* model. These include expression of tight junction proteins, polarized expression of multiple ABC/SLC transporters (including P-glycoprotein, breast cancer resistance protein and organic cation transporters [19,20]. The immortalised mouse brain endothelial (bEnd.3) cells are a commercially available mouse BBB cell line and are established from the SV129 strain. The expression of various BBB specific transporters have been determined in bEnd.3 [21]. These two well-established *in vitro* models of the adult BBB were utilised.

All hCMEC/D3 cells used here were between passages 25–35. hCMEC/D3 cells were grown using the Clonetics^®^ EGM^®^- 2 MV Bullet Kit (Lonza, Wokingham,UK), containing the endothelial cell medium ‘EBM-2’ (500 ml) and the growth factor kit SingleQuots™ containing insulin-like growth factor-1, vascular endothelial growth factor, epidermal growth factor, hydrocortisone and basic fibroblast growth factor according to the manufacturer’s instructions and as previously described [19,20]. The cells were grown in 75 cm^2^ flasks (T-75) or 96 well plates pre-coated with rat tail collagen type 1 (Gibco, Invitrogen) at a concentration of 0.1 mg/ml for 2 hours at 37°C and then washed with phosphate buffered saline with calcium and magnesium and no phenol red (referred to as PBS+) to remove suspended collagen. The cells were grown in an incubator with a saturated humidity at 37°C in 5% CO_2_ and 95% fresh air. They were split when they reached 80–90% confluency (reached in 3–4 days) and seeded at a density of 25,000 cells/cm^2^ in 96-well plates for experiments. The cells grown on 96 well plates were left for further 3–4 days to completely differentiate into brain endothelial cells before carrying out the experiments. Media was changed every 2–3 days.

bEnd.3 cells were maintained in DMEM (D6429 from Sigma, UK) + 10% foetal bovine serum (FBS) and 1% Penicillin-Streptomycin. Cells were split with trypsin before reaching 90% confluency, usually every 3–4 days. The cells were seeded onto a T-75 and 96 well plates for experiments at a density of 20,000 cells/cm^2^. All cells used were between passages 19–28.

### Drug accumulation assay

Experiments were carried out on a monolayer of hCMEC/D3 or bEnd.3 cells grown on the centre 60 wells of 96-well plates. To determine the accumulation of the [^3^H]pentamidine in the cells, the culture medium was removed and the cells in each well were incubated with 200μl of the accumulation buffer (Table A in S1 File) for composition of the buffer) along with [^3^H]pentamidine (9 nM) and [^14^C]sucrose (0.9 μM) which acts as a marker for non-specific binding, extra cellular space, and barrier integrity. Control conditions included [^3^H]pentamidine, [^14^C]sucrose and 0.05% DMSO. The cells were exposed to [^3^H]pentamidine in the accumulation buffer for 5, 20, 30, 60, and 120 minutes. The accumulation assays were carried out on a temperature-controlled shaker (THERMOstar, BMG Labtech, Offenburg, Germany) set at 37°C and 120 rpm. After the exposure, the buffer was aspirated and the wells were washed three times with ice-cold PBS+ (Sigma Aldrich, UK) to remove the radiolabelled drug that was not taken up by cells and to stop any further transport into the cells. 200 μl of 1% Triton x-100 (Sigma, UK) was then added to each well and the plate was incubated for 1 hour in a temperature-controlled shaker at 37°C to lyse the cells and to release the radiolabelled drug. 100 μl from each of the wells was then transferred to a scintillation vial with 4ml of scintillation fluid (Optiphase Hisafe 2, PerkinElmer, UK). The remaining 100 μl was used to carry out a BCA^™^ protein assay to determine the protein concentrations in each well, using bovine serum albumin (BSA) as standards, and measured spectrophotometrically at 562 nm on a Labsystems Multiscan reader with Ascent software.

The radioactivity was determined using the Packard Tri-Carb 2900TR liquid scintillation counter (Perkin-Elmer, Beaconsfield, UK). The sum of accumulated radioactivity (a sum of efflux and influx of the compound) was calculated to find the volume of distribution (V_d_). It was determined from the ratio of dpm/mg protein to dpm/μl buffer. Background radiation (dpm for accumulation buffer alone without radioactive drugs) and V_d_ values for [^14^C]sucrose were used to correct the V_d_ values for [^3^H]pentamidine [20,22]. All graphs presented in this paper have been corrected for sucrose.

### Transporter inhibition assay

The presence of a saturable transport mechanism was investigated by comparing the accumulation of radiolabelled pentamidine in the absence and presence of 10 μM unlabelled pentamidine. The BBB expresses the ATP-dependent binding cassette (ABC) transporters, P-gp, BCRP, and MRPs which are involved in the efflux of compounds from the brain capillary endothelial cell into the blood [23]. To assess the role of ATP-dependent mechanisms in the transport of pentamidine, ATP was removed from hCMEC/D3 and bEnd.3 cell lines by incubating the cells with 10 mM 2-DG (Sigma, UK), as previously described [20]. Substrates and inhibitors of these transporters were also used to assess transporter involvement of pentamidine delivery across the BBB. See Table 1 for further details. In addition the involvement of specific uptake transporters which are known to be expressed at the brain capillary endothelial membrane such as the OCT/OCTNs (SLC22A family), MATE (SLC47 family) and PMAT (SLC29 family), which selectively transport cationic organic molecules, concentrated nucleoside transporters (CNTs, SLC28 family) and equilibrative nucleoside transporters (ENTs, SLC29 family) which transport nucleoside and nucleobase compounds [24,25] were studied.

**Table 1:** The transporter inhibitors used in this study along with [^3^H]pentamidine. All inhibitors were used in the presence of 0.05% DMSO and used at the published concentration ranges where they affect transporter activity.

Target Transporter	Transporter Inhibitor/substrate	Concentration	Source	References
-	Pentamidine isethionate salt	10 μM	Sigma, UK	[20]
P-gp	Haloperidol	40 μM	Sigma, UK	[20,26]
P-gp	Dexamethasone	200 μM	Sigma, UK	[20]
BCRP	ko143	1 μM	Tocris Bioscience, UK	[20,26]
BCRP	Pheophorbide A	1 μM	Santa Cruz Biotechnology, Germany	[20]
MRPs	MK571	10 μM	Cambridge Bioscience, UK	[19]
OCT1 and 3	Prazosin	100 μM	Sigma, UK	[13]
OCT1 and 2	Amantadine	500 μM	Sigma, UK	[13]
OCT2	N-methylnicotinamide	100 μM	MP Biomedicals Europe, UK	[13]
OCT3	Corticosterone	50 μM	Sigma, UK	[13]
OCTN1	Ergothioneine	20 μM	Insight Biotechnology, UK	[27]
OCTN2	L-carnitine	5 μM	Insight Biotechnology, UK	[28]
MATE1	Famotidine	1 μM	Insight Biotechnology, UK	[29]
MATE2	Nifekalant	3 μM	Insight Biotechnology, UK	[29]
PMAT	Lopinavir	2 μM	Insight Biotechnology, UK	[30]
ENTs and CNTs	Adenosine	100 μM	Sigma, UK	[31][32]
CNT1 and 2	Fludarabine	50 μM	Cambridge Bioscience, UK	[24]

### (3-(4,5-Dimethylthiazol-2-yl)-2,5-diphenyltetrazolium bromide (MTT) cytotoxicity assay

This assay was carried out as previously described by Watson et al. (2012) [20]. In summary, the assay is based on the yellow tetrazole, (3-(4,5-dimethylthiazol-2-yl)-2,5-diphenyltetrazolium bromide (MTT) (Life Technologies, UK) which is transformed into a purple formazan by a mitochondrial enzyme[33]. Therefore, only viable cells are able to carry out this reaction. In brief, inhibitor (Table 1) accumulation studies (without radiolabelled drugs) were performed and 200 μl of accumulation buffer removed at 120 minutes. Then 100 μl of 1 mg/ml MTT in DMEM without phenol red (Life Technologies, UK) was added to each well. The plates were then incubated for 4 hours at 37°C. After the incubation, the solution was removed; 100 μl 100% propan-2-ol was added to each well and the absorbance values measured. Protein content was measured using BCA assay and the absorbance values were corrected for the protein content. The viability of cells was expressed in percentage compared to control cells. Our group have previously published the MTT assay results on hCMEC/D3 cells [20] for some of the inhibitors/substrates used in this study. The compounds not previously tested in hCMEC/D3 cells and all compounds used on bEnd.3 cells were tested for their cytotoxic effects here.

### Transmission electron microscopy (TEM)

Endothelial cell morphology was investigated using transmission electron microscopy (TEM) and the hCMEC/D3 and the bEnd.3 cells were found to grow as a confluent monolayer with the culture conditions described (Figs A and B in S1 File). For TEM analysis, cells grown on transwell filters were fixed overnight at 4°C in 2.5% (v/v) glutaraldehyde in 0.1 M cacodylate buffer (pH 7.3) and post-fixed in 1% (w/v) osmium tetroxide for 1.5 hours at 4°C. After washing, samples were then dehydrated through a graded ethanol series before infiltration with TAAB epoxy resin for 4 hours at room temperature. Filter pieces were cut using a razor blade, embedded on flat moulds and polymerised at 70°C for 24 hours. Ultrathin sections (50–70 nm) were prepared using a Reichert-Jung Ultracut E ultramicrotome, mounted on 150 mesh copper grids and contrasted using uranyl acetate and lead citrate. Samples were examined on a FEI Tecnai 12 transmission microscope operated at 120 kV. Images were acquired with an AMT 16000M camera.

For TEM immunogold labelling, hCMEC/D3 cells were grown on collagen-coated transwell filters (Corning^®^ Transwell^®^ polycarbonate membrane cell culture inserts) and bEnd.3 cells (used as a negative control for the primary antibody) were grown on transwell filters without any coating. The membranes were lightly fixed with 4% paraformaldehyde / 0.1% glutaraldehyde in 0.1 M phosphate buffer pH 7.2. After fixation, filters were thoroughly rinsed with phosphate buffer, cut out of the plastic well frame and flat embedded in 12% gelatine in PBS which was sandwiched between 2 glass slides. Gelatine blocks including small portions of the filters (1–2 mm cubes) were selected for further processing and cryoprotected by incubation in 2.3 M sucrose overnight at 4°C. After cryoprotection, samples were mounted on aluminium pins and cryofixed by plunging into liquid nitrogen. Samples were stored in liquid nitrogen prior to cryosectioning. Ultrathin sections (70-90nm thick) were cut using a Leica EM FC6 cryoultramicrotome (Leica Microsystems UK, Ltd) and mounted on pioloform film-supported nickel grids by the Tokuyasu method[34]. Sections were immune-labelled with anti-OCT1 primary antibody (Novus Biologicals, NBP1-51684) at 1:500 followed by 12 nm-colloidal gold secondary antibody (Jackson ImmunoResearch, Europe, UK) at 1:40 and then examined using an FEI Tecnai 12 transmission microscope operated at 120 kV. Images were acquired with an AMT 16000M digital camera. To quantify labelling, 60 sequential images of the cell monolayer were taken at a magnification of 11500x and the number of dots (labels) counted in these images to calculate average number of nanoparticles per frame.

### Animals and tissue collection

All procedures were performed within the guidelines of the Animal Scientific Procedures Act (1986) and Amendment Regulations 2012. The study was approved by the King’s College London Animal Welfare and Ethical Review Body. BALB/c mice were purchased from Harlan UK Limited (Bicester, Oxon, UK). All animals were maintained under standard temperature/lighting conditions and given food and water ad libitum.

### Brain capillary isolation for Western blotting

Brain capillaries were isolated from wild-type Balb/c mice (n = 2). Adult male mice (∼25 g) were anaesthetised (2 mg/kg i.p. medetomidine hydrochloride and 150 mg/kg i.p. ketamine) and heparinized (100 U i.p.). The left ventricle of the heart was cannulated and perfused (5 ml/min) with oxygenated artificial plasma (Table B in S1 File) for up to 2 minutes. The right atrium was sectioned before perfusion was started. At the end of the time, the mice were decapitated and the perfused brain removed. The brain was then homogenised in physiological buffer (brain weight × 3) and 26% dextran (brain weight × 4) (Table C in S1 File). The homogenate was subjected to density gradient centrifugation (5,400 × g for 15 min at 4°C) to give an endothelial cell-enriched pellet and the supernatant was discarded [2].

### SDS-PAGE and Western blot

Western blot (WB) was carried out to determine the expression of all the transporters that were targeted by the inhibitors used. To analyse cells, confluent layer of hCMEC/D3 and bEnd.3 were grown in a T-75 and lysed using Radio-Immunoprecipitation Assay (RIPA) buffer from Sigma-Aldrich Company, UK and 30 μg of protein was loaded per well. The positive control was provided by an endothelial cell enriched pellet obtained from capillary depletion analysis of an adult Balb/c mouse brain which had been perfused as described above. Novex^®^ 4–20% Tris-Glycine Mini Gels (Life Technologies, UK) were used. After electrophoresis, the protein was transferred onto a methanol activated Immobilon-P PVDF membrane using the wet transfer method. The membrane was then blocked for an hour using 5% skimmed milk powder at room temperature (RT). After extensive washing in PBS-Tween, the membrane was then incubated with the specific primary antibody (Table D in S1 File) overnight at 4°C. The following day the membrane was washed again 3 x with PBS-Tween and incubated for an hour at RT with the specific secondary antibody conjugated with horseradish peroxidase (HRP). After the washing step with PBS-T, the membrane was visualised using enhanced chemiluminescence using Genesnap G:box and software (Syngene) to visualise the protein bands [35][36].

### Confocal microscopy (Immunofluorescence, IF)

Cells were grown on 0.4 μM polyester transwell^®^ filters which were coated with rat-tail collagen type 1 for hCMEC/D3 cells. There was no coating for bEnd.3 cells. Cells were incubated with the cell membrane marker tetramethylrhodamine conjugated Wheat Germ Agglutinin (WGA) for one hour at 4°C and then fixed with 4% paraformaldehyde for 20 minutes at RT. After washing with PBS^+^, they were incubated with 0.1% Triton X-100 for 5 minutes to permeabilise the membranes. They were then incubated with primary antibody in 10% goat-serum in PBS^+^ overnight at 4°C. The following day, after washing with PBS^+^, they were incubated with secondary antibody for one hour at RT (Table D in S1 File). Following secondary incubation, cells were washed with PBS^+^ and the filters cut out from the holders and mounted on microscope slides using Vectashield mounting medium with DAPI (Vector Laboratories Ltd, Peterborough, UK). Confocal studies were conducted using Eclipse Ti-E Live Cell Imaging System 1 with C2+ confocal system using NIS Elements AR software (Nikon Imaging Centre, King’s College London).

### Data analysis and Statistics

Data are all expressed as mean ± SEM unless stated otherwise. Data were analysed by one-way ANOVA with Tukey's post hoc test, two-way ANOVA with Holm-Sidak post-hoc test or a paired t-test using Sigmaplot version 13 from Systat Software, Inc., San Jose California USA.

## Results

### Pentamidine accumulation studies

In all accumulation experiments the [^14^C]sucrose values were not significantly different to those where there was a test inhibitor or substrate present except for prazosin (100 μM) in bEnd.3 cells only.

### ATP depletion and self-inhibition

**Fig 1:**
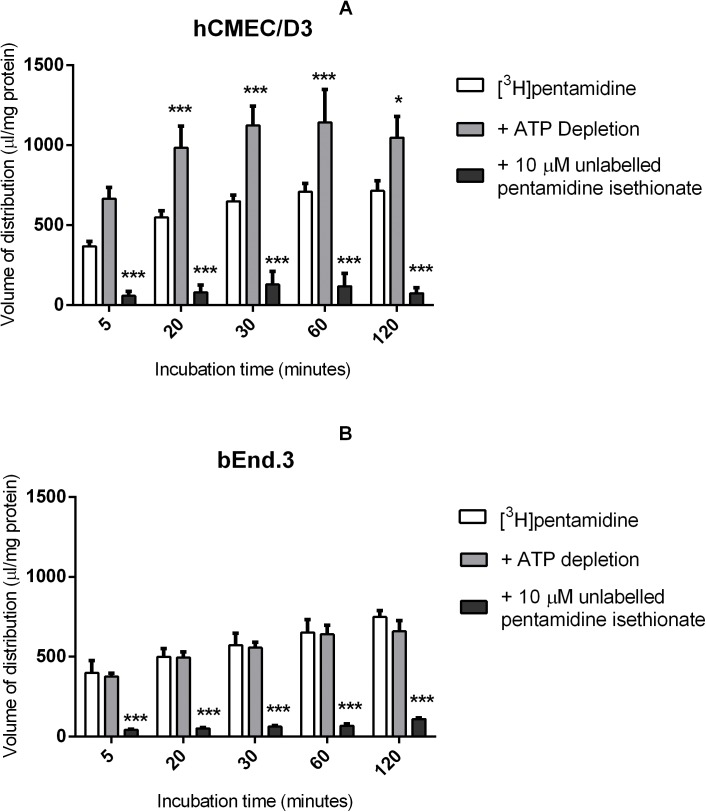
The effect of ATP depletion and self-inhibition on [^3^H]pentamidine accumulation was determined in hCMEC/D3s (A) and bEnd.3s (B). Significant differences compared to control was observed—*p<0.05, **p<0.01, ***p<0.001. All data expressed as mean ± SEM, n = 4 passages of cells with 6 replicates (wells) per timepoint per plate. Data were analysed using two-way ANOVA with SigmaPlot 13.0.

ATP depletion caused a significant increase in [^3^H]pentamidine accumulation in hCMEC/D3 at all time points except 5 minutes. On average [^3^H]pentamidine accumulation increased by 65% compared to control when ATP was removed (Fig 1A, *p<0.05). However, ATP depletion had no effect on [^3^H]pentamidine accumulation in bEnd.3 cell lines (Fig 1B).

[^3^H]pentamidine (9 nM) was also incubated with 10 μM unlabelled pentamidine isethionate to determine if pentamidine is transported into the cells and if this transport is saturable i.e. using a transporter. The results show that compared to control conditions, accumulation of [^3^H]pentamidine in both hCMEC/D3 and bEnd.3 cells decreased significantly (Fig 1) (by 89% for hCMEC/D3 cells and 85% for bEnd.3 cells compared to control after 120 minutes) (***p<0.001).

### The role of ABC transporters

**Fig 2:**
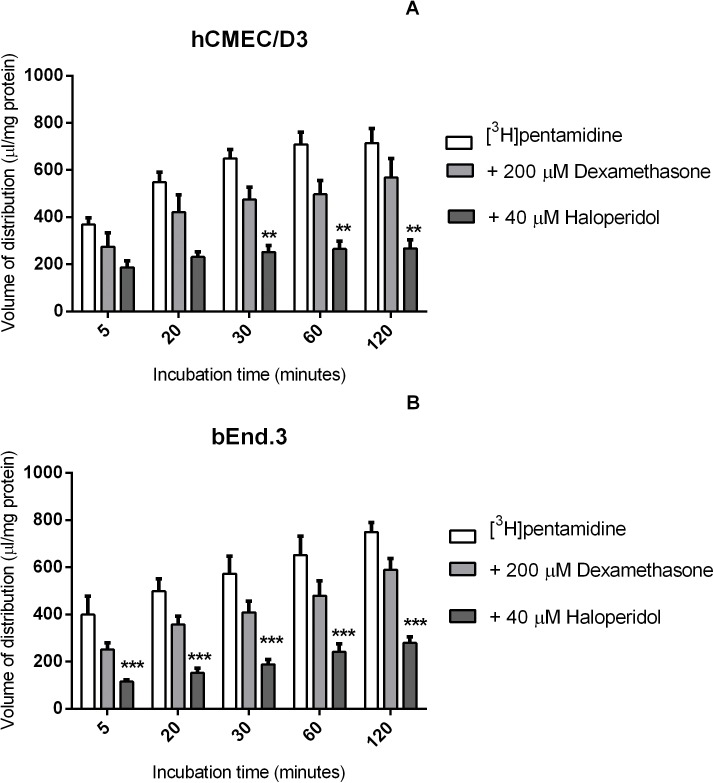
The effect of P-gp substrates and inhibitors on the [^3^H]pentamidine accumulation in hCMEC/D3 (A) and bEnd.3 (B) cells. Cells were incubated with P-gp substrate dexamethasone (200 μM) or P-gp inhibitor haloperidol (40 μM) and no significant differences were observed compared to control. All data expressed as mean ± SEM, n = 4 passages of cells, with 6 replicates (wells) per timepoint per plate. Data were analysed using two-way ANOVA with SigmaPlot 13.0,

[^3^H]pentamidine accumulation was studied in the presence of a P-gp substrate (dexamethasone, 200 μM) and a P-gp inhibitor (haloperidol, 40 μM) to understand the role of P-gp in pentamidine transport (Fig 2). The results showed that there was no effect of dexamethasone on [^3^H]pentamidine accumulation in both the hCMEC/D3 and the bEnd.3 cell lines. However, haloperidol significantly decreased the accumulation of [^3^H]pentamidine in both cell lines (**p<0.01 for hCMEC/D3 and ***p<0.001 for bEnd.3). For hCMEC/D3 cells, the accumulation was decreased by 62% after 120 minutes and for bEnd.3 cells, the accumulation was decreased by 66% after 120 minutes compared to control (Fig 2).

**Fig 3:**
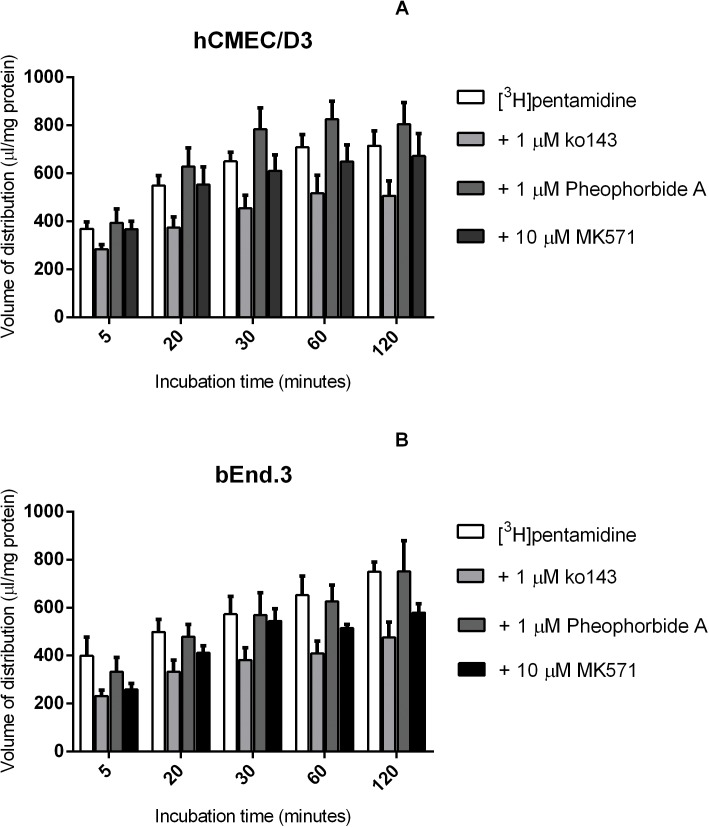
The effect of BCRP and MRP inhibitors on [^3^H]pentamidine accumulation in hCMEC/D3 (A) and bEnd.3 (B) cells. Cells were incubated with BCRP inhibitor ko143 (1 μM), BCRP substrate pheophorbide A (1 μM) or MRP inhibitor MK571 (10 μM) and no significant differences were observed compared to control. All data expressed as mean ± SEM, n = 4 passages of cells, with 6 replicates (wells) per timepoint per plate. Data were analysed using two-way ANOVA with SigmaPlot 13.0.

Incubation of cells with the BCRP inhibitor, ko143 (1 μM), the BCRP substrate pheophorbide A (1 μM), and the inhibitor for the MRP family of transporters, MK571 (10 μM) with [^3^H]pentamidine and [^14^C]sucrose did not significantly change the accumulation of [^3^H]pentamidine inside the cells (Fig 3).

### The role of OCT transporters

**Fig 4:**
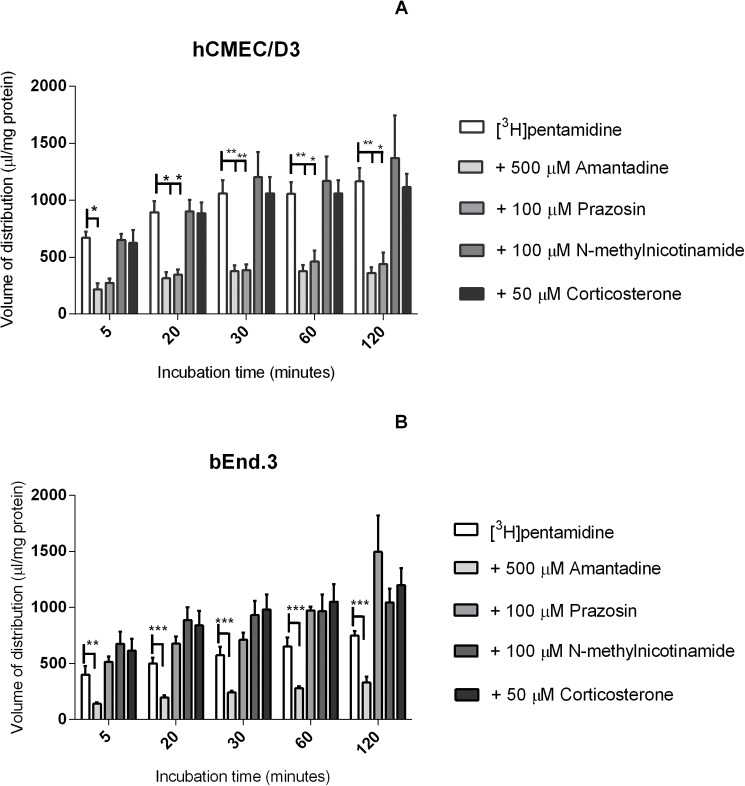
Influence of OCT 1, 2, and 3 transporters on [^3^H]pentamidine accumulation were studied on hCMEC/D3(A) and bEnd.3 (B) cell lines. Cells were incubated with amantadine (500 μM, OCT1 and 2 inhibitor), prazosin (100 μM, OCT1 and 3 inhibitor), N-methylnicotinamide (100 μM, OCT2 inhibitor) or corticosterone (50 μM, OCT3 inhibitor). Significant differences were observed compared to control—*p<0.05, **p<0.01, ***p<0.001. All data expressed as mean ± SEM, n = 3–4 passages of cells, with 6 replicates (wells) per timepoint per plate. Data were analysed by two-way ANOVA with SigmaPlot 13.0.

The OCT1 and OCT2 inhibitor, amantadine (500 μM), and the OCT1 and OCT3 inhibitor prazosin (100 μM), were incubated with [^3^H]pentamidine and [^14^C]sucrose. A significantly decreased accumulation of pentamidine was observed in both cell lines with amantadine; amantadine decreased pentamidine accumulation by 45% on average compared to control in hCMEC/D3 cells (**p< 0.01) and by 59% on average in bEnd.3 cells (***p< 0.001) after 120 minutes. Prazosin decreased pentamidine accumulation by 39% in hCMEC/D3 cells (*p< 0.05) but no decrease was observed with bEnd.3 cells. Prazosin also significantly increased [^14^C]sucrose values after 2 hours in bEnd.3 cells only (*p< 0.05). Incubation with N-methylnicotinamide (100 μM, OCT2 inhibitor) and corticosterone (50 μM, OCT3 inhibitor) did not lead to any significant effects (Fig 4).

### The role of OCTN1 and OCTN2 transporters

**Fig 5:**
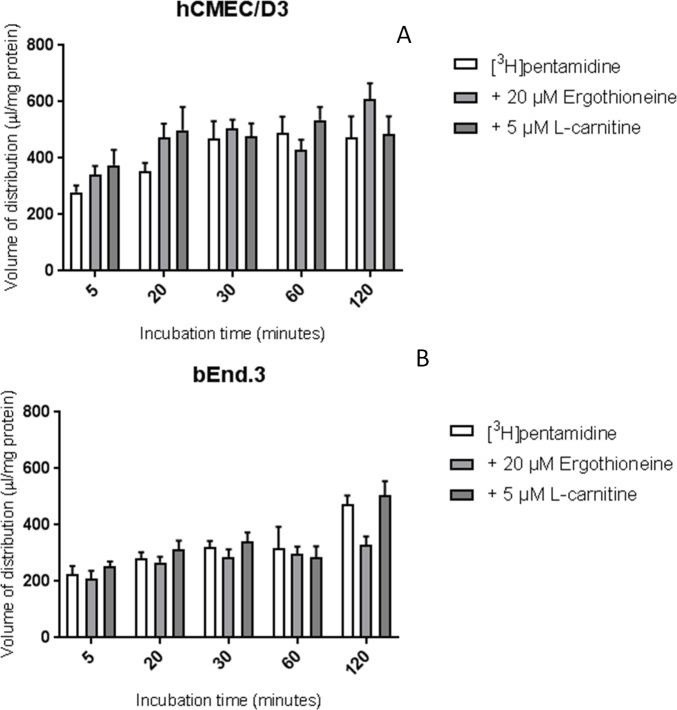
The effect of OCTN1 and OCTN2 substrates on the [^3^H]pentamidine accumulation in hCMEC/D3 (A) and bEnd.3 (B) cells. Cells were incubated with OCTN1 substrate ergothioneine (20 μM) or OCTN2 substrate L-carnitine (5 μM) and no significant differences were observed compared to control. All data expressed as mean ± SEM, n = 4 passages of cells, with 6 replicates (wells) per timepoint per plate. Data were analysed using two-way ANOVA with SigmaPlot 13.0.

Incubation with OCTN1 substrate, ergothioneine (20 μM), and the OCTN2 substrate L-carnitine (5 μM), did not significantly affect the accumulation of [^3^H]pentamidine in either cell line (Fig 5).

### The role of MATE1, MATE2 and PMAT transporters

**Fig 6:**
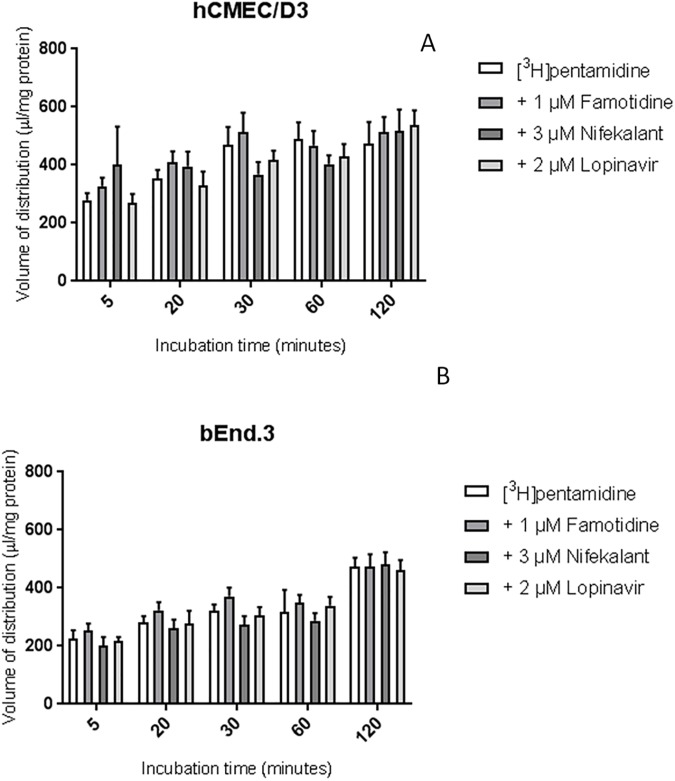
The effect MATE1, MATE2 and PMAT inhibitors on the [^3^H]pentamidine accumulation in hCMEC/D3 (A) and bEnd.3 (B) cells. Cells were incubated with MATE1 inhibitor famotidine (1 μM), MATE2 inhibitor nifekalant (3 μM) or PMAT inhibitor lopinavir (2 μM) and no significant differences were observed compared to control. All data expressed as mean ± SEM, n =.4 passages of cells, with 6 replicates (wells) per timepoint per plate. Data were analysed using two-way ANOVA with SigmaPlot 13.0.

Incubation with MATE1 inhibitor, famotidine (1 μM), MATE2 inhibitor, nifekalant (3 uM) and PMAT inhibitor, lopinavir (2 μM) did not significantly affect the accumulation of [^3^H]pentamidine in either cell line (Fig 6).

### The role of ENT and CNT transporters

Incubation with the ENT1 and CNT2 substrate, adenosine (100 μM), and the CNT1 and 2 inhibitor, fludarabine (50 μM), did not significantly affect the accumulation of [^3^H]pentamidine in either cell line (Fig C in S1 File).

### MTT cytotoxicity assay

**Fig 7:**
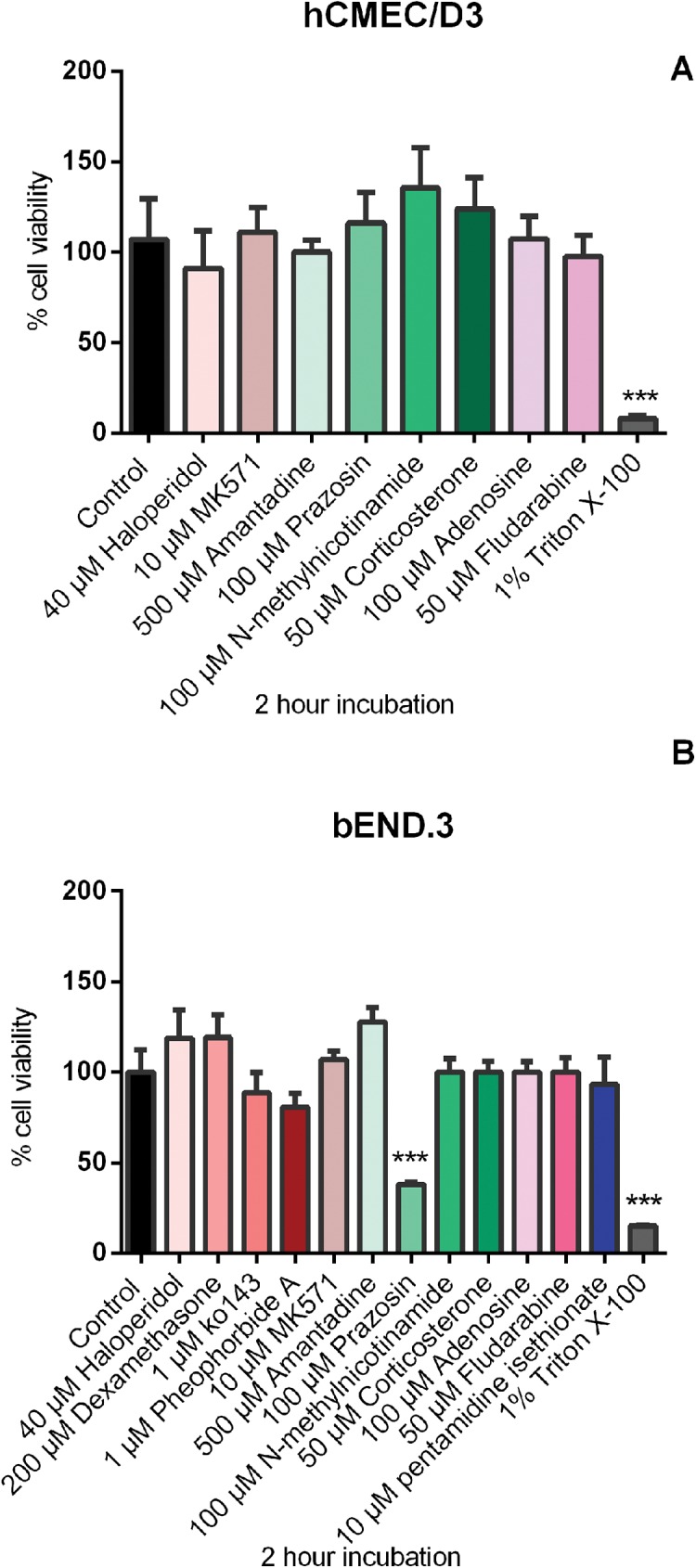
MTT assay was carried out to assess the cytotoxic effects of compounds used by incubating the compounds on confluent monolayers of hCMEC/D3 (A) and bEnd.3 (B) cells. Cells grown in 96-well plates were exposed to the inhibitor containing buffer for 120 minutes. Results are expressed as percentage viability of cells ± SEM compared to control where cells were incubated with accumulation buffer alone. A positive control of 1% Triton X-100 was also included. Significant differences compared to control was observed—*p<0.05, **p<0.01, ***p<0.001. All data expressed as mean ± SEM, n = 6 wells. Data were analysed using one-way ANOVA with Sigma Plot 13.0.

MTT assays were carried out to assess the potential cytotoxic effects of the test substrates and inhibitors used in this study. The control group were cells treated with accumulation buffer alone in the absence of test molecules. The cells were exposed to the inhibitors/transporters for 2 hours. The majority of test molecules did not significantly affect cell viability. The exception was prazosin (100 μM) which caused a 60% decrease in bEnd.3 cell viability (***p<0.001) but had no effect on hCMEC/D3 cells. 1% triton X-100 (positive control) decreased cell viability by 90% in both cell lines (***p<0.001, Fig 7).

### Expression of transporters

#### ABC transporter expression

**Fig 8:**
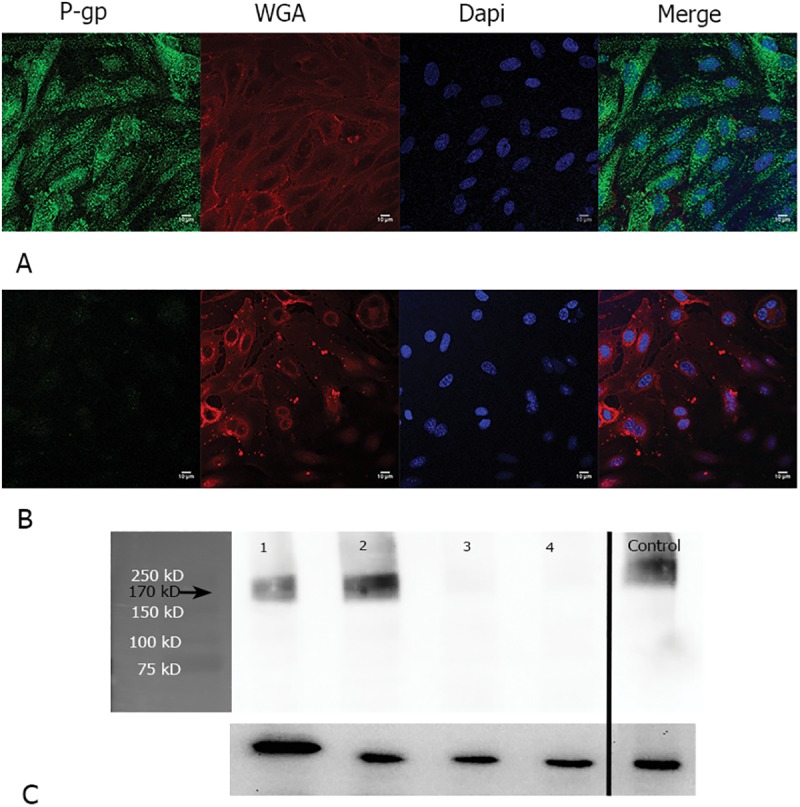
P-gp expression in confluent hCMEC/D3 cells and bEnd.3 cells. Confocal microscopy image of P-gp expression in confluent hCMEC/D3 cells (A) and bEnd.3 cells (B) was detected using anti-P-gp antibody (Abcam, ab170904) diluted at 1:200. Western blot band of P-gp was detected at 170 kD after probing with anti-P-gp antibody (Abcam, ab170904) diluted at 1:2000. Tubulin (55 kD) was used as a loading control. (C)—Lane 1 –hCMEC/D3 passage 28, Lane 2—hCMEC/D3 passage 33, Lane 3- bEnd.3 passage 18, Lane 4 –bEnd.3 passage 23, and MDCK-hMDR (positive control from the same gel).

ABC transporter expression was determined by Western blotting and confocal microscopy in both cell lines. P-gp expression was positive in hCMEC/D3 cells but negative in bEnd.3 cells as clearly observed by both confocal microscopy (Fig 8A and 8B) and Western blotting (Fig 8C).

**Fig 9:**
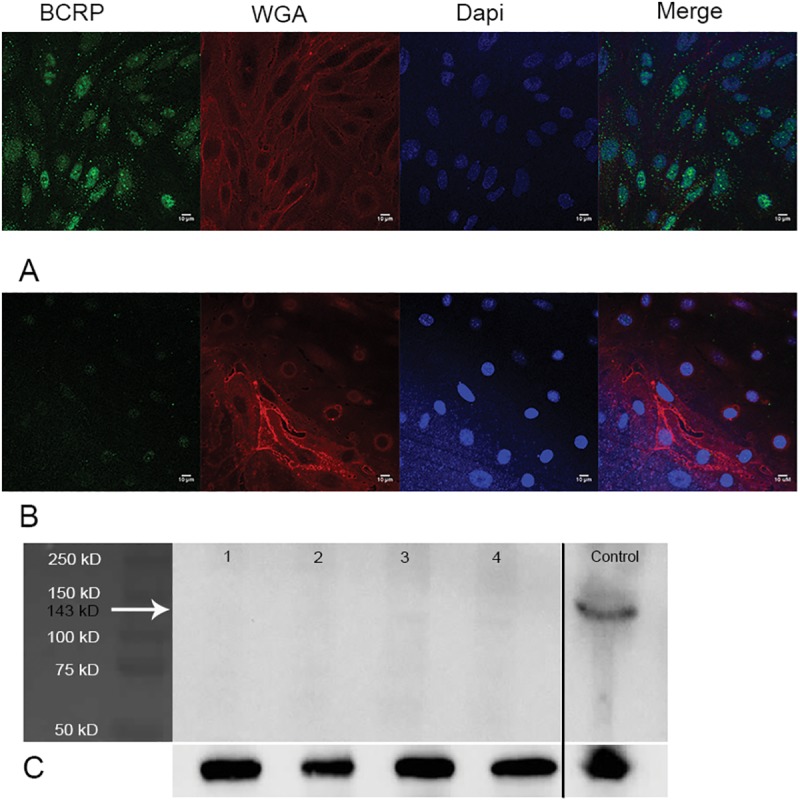
BCRP expression in confluent hCMEC/D3 cells and bEnd.3 cells. Confocal microscopy image of BCRP expression in confluent hCMEC/D3 cells (A) and bEnd.3 cells (B) was not detected in the plasma membranes when using anti-BCRP antibody (New England Biolabs, 4477S diluted at 1:200)—Western blot band of BCRP was detected at 143 kD which corresponds to the dimer version of BCRP in positive control (C). No band was observed at 70 kD (monomer version of BCRP). Tubulin (55 kD) was used as a loading control. Lane 1 –hCMEC/D3 passage 28, Lane 2- hCMEC/D3 passage 33, Lane 3- bEnd.3 passage 18, Lane 4- bEnd.3 passage 23, Lane 5 –brain endothelial cells isolated from mouse (positive control).

BCRP expression was not detectable in the plasma membranes of hCMEC/D3 cells or bEnd.3 cells as observed by confocal microscopy and Western blotting. However, Western blotting revealed that brain endothelial cells isolated by capillary depletion from mice (Balb/c) expressed a band at 143 kD representing the homo-dimer version of BCRP [37] (Fig 9). Confocal microscopy using another BCRP antibody (BXP-21; Ab3380) confirmed the absence of BCRP in hCMEC/D3 cells (Fig D in S1 File).

**Fig 10:**
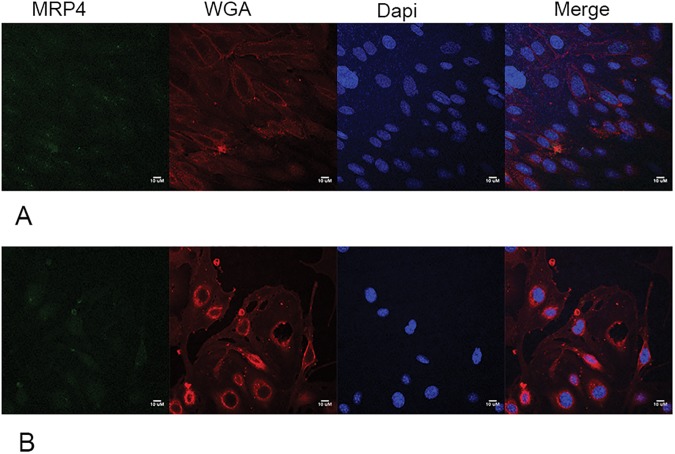
Confocal microscopy image of MRP4 expression in confluent hCMEC/D3 cells (A) and bEnd.3 cells (B). No fluorescent signals were detected when using MRP4 antibody [M4I-10] from Abcam at 1:200 dilution and goat anti-rat Alexa Fluor^®^ 488 secondary antibody (Abcam, ab181448) at 1:200 dilution.

MRP4 transporter expression levels were below detectable levels in both cell lines (Fig 10).

#### OCT transporter expression

**Fig 11:**
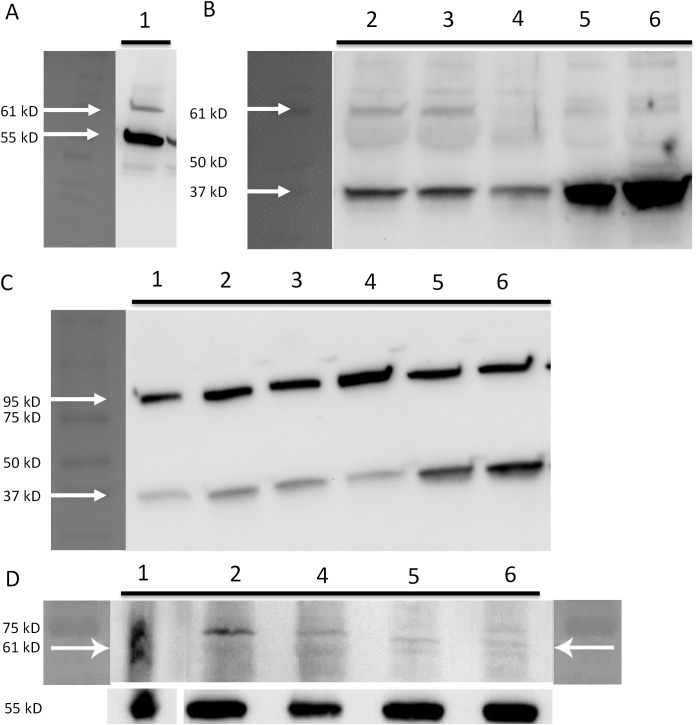
OCT expression in confluent bEnd.3 cells and hCMEC/D3 cells. **A)** OCT1 expression observed in brain endothelial cells isolated from mouse (positive control) at 61 kD when using anti-OCT1 antibody (Abcam, ab55916) at 1:750 dilution. **B)** OCT1 bands were also observed at 61kD in 2,3,5 and 6. **C)** OCT2 expression was observed in all cell lines at ~ 95 kD when using anti-OCT2 antibody (Abcam, ab170871) at 1:2000 dilution in accordance to the datasheet. **D)** OCT3 expression was observed in all cell lines at 61 kD when using anti-OCT3 antibody (Abcam, ab183071) at 1:600 dilution. Tubulin expression was used as a loading control in **A** and **D** at 55 kD when using anti-tubulin antibody (Millipore Limited, UK) at 1:10000 dilution. GAPDH expression was used as a loading control in **B** and **C** at 37 kD when using anti-GAPDH antibody (Abcam, ab9485) at 1:2500 dilution. 1 –Brain endothelial cells isolated from mouse (positive control), 2—bEnd.3 passage 18, 3 –bEnd.3 passage 19, 4 –bEnd.3 passage 23, 5—hCMEC/D3 passage 28, 6—hCMEC/D3 passage 33.

OCT1 (SLC22A1), OCT2 (SLC22A2) and OCT3 (SLC22A3) expression was observed using Western blots in both hCMEC/D3 and bEnd.3 cell lines at early and late passages (Fig 11).

**Fig 12:**
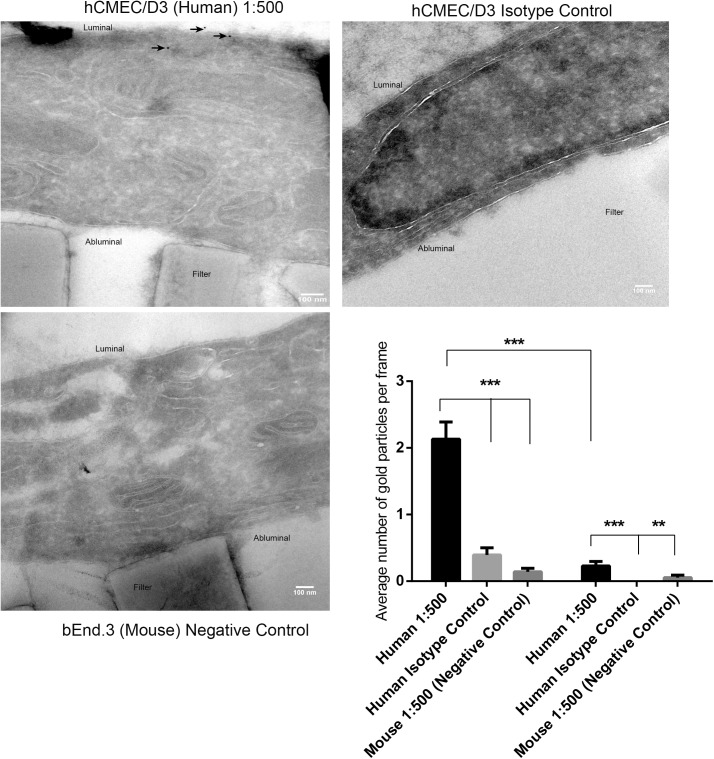
Expression of OCT1 was detected by Transmission Electron Microscopy and immunogold labelling. hCMEC/D3 (p28) and bEnd.3 cells (p23) were grown to confluency on transwell polycarbonate membrane and stained for OCT1 using the method described. Gold nanoparticles on the membranes (arrows) were counted in the 60 sequential images acquired at 11500 x magnification. Significantly increased expression of OCT1 was found on the luminal membrane compared to the abluminal membrane. ***p<0.001 using student’s t-test.

TEM images were acquired for hCMEC/D3 cells to determine the localisation of OCT1 using immunogold staining (Fig 12). Immunolabelling was detected both in the cytoplasm (31%) and on the membranes (69%). Within the membranes, there was significantly more expression of OCT1 in the luminal membrane compared to the abluminal membrane (***p<0.001).

## Discussion and conclusions

Pentamidine, the first line treatment for stage 1 HAT, is an effective drug against *T*.*b*. *gambiense* with a cure rate of 94% for early-late stage HAT [38]. Therefore, it is vital to understand why pentamidine has limited ability to cross cell membranes, particularly, the highly specialised cerebral capillary endothelium known as the BBB. Interestingly, Sanderson et al. (2009) [2] noted that multiple transporters existed for pentamidine at the BBB including mouse mdr1a/mdr1b (P-glycoprotein). In addition, it was observed that pentamidine accumulated inside the brain endothelial cells significantly more than it reached the brain parenchyma, suggesting that transport of pentamidine from the blood into the BBB is more efficient than it is from the BBB into the brain. In light of this study, the current investigation was undertaken with the aim of understanding the limited permeability of pentamidine across the human and mouse BBB in more detail and to identify the transporters involved.

The limited ability of pentamidine to enter the mouse brain was previously shown to be partly the result of efflux from the BBB into the blood by ABC transporters [2]. ABCs are a set of highly efficient neuroprotective transporters responsible for limiting the brain penetration of potentially neurodamaging xenobiotics. They use ATP as an energy source to drive substrates from the endothelial cell back into the blood[39]. Here, ATP depletion assays were carried out which resulted in significant increase in [^3^H]pentamidine accumulation in hCMEC/D3 cells, but not bEnd.3 cells. This difference between the cell lines could be related to the significant presence of P-gp detected in the hCMEC/D3s but not bEnd.3s according to Western blots and confocal microscopy images. The lack of P-gp expression in bEnd.3 found here is in contrast to the study by Park et al. (2014) [21] who found significant P-gp expression in bEnd.3s. This could be the result of differences in culture conditions between laboratories[40].

Functional ABC transporter activity was also assessed in this present study by targeting individual transporters with specific inhibitors or substrates. No effects were observed with P-gp substrate dexamethasone, the BCRP inhibitor, ko143, and the substrate pheophorbide A and the MRP family inhibitor, MK571 on [^3^H]pentamidine accumulation in either cell line. MRP4 was not observed in either cell line which could be why MK571 did not have any observable effects on radiolabelled pentamidine accumulation. Here, the lack of visible effects after inhibiting individual efflux transporters is probably because other efflux transporters could functionally replace the inhibited transporter. It is also possible that the inhibitors used are unable to block the specific transporter completely and/or have a different binding site leading to an apparent lack of effect.

The present study provides functional evidence for an ATP-dependent mechanism of efflux that is responsible for the transport of pentamidine from the endothelial cell back into the blood. This is in agreement with previous results from our laboratory [2].

Following this set of experiments, we investigated how [^3^H]pentamidine is taken up into the endothelial cell; both cell lines were incubated with unlabelled pentamidine isethionate. This resulted in a significant decrease in the accumulation of [^3^H]pentamidine inside the cells suggesting the presence of a transporter responsible for taking the drug into the endothelial cells.

Interestingly, incubation with haloperidol resulted in a significant decrease in [^3^H]pentamidine accumulation suggesting haloperidol interaction with an uptake transporter of pentamidine rather than P-gp. The significant decrease in the accumulation of [^3^H]pentamidine when haloperidol was present was surprising as haloperidol is considered to be a P-gp inhibitor[41] [. However, haloperidol has been identified as an OCT substrate in Human Embryonic Kidney (HEK) cell line over-expressing OCT1 since haloperidol was able to inhibit the uptake of OCT1 substrate 4-(4-(dimethylamino)styryl)-N-methylpyridinium (ASP+) by 67% [42]. In addition, haloperidol metabolites have been shown to interact with OCTs in Caco-2 cells [43]. Accumulation of these metabolites when the Caco-2 cells were pretreated with hOCT inhibitors decreased significantly.

Pentamidine, like haloperidol, is a positively charged compound. It has a physiological charge of +2 and therefore hypothesized to be a substrate for the OCTs. OCT1 and 3, but not OCT2, were shown to be expressed in both cells lines by Western blotting. Thus, hCMEC/D3 and bEnd.3 cells were treated with amantadine, prazosin, corticosterone, and N-methylnicotinamide, which are known OCT inhibitors/substrates. Results show that amantadine significantly decreased the accumulation of [^3^H]pentamidine in both cell lines, however, prazosin only had significant effects on hCMEC/D3 cells. This could be because over the duration of the experiment prazosin was found to be toxic to bEnd.3 cells and not hCMEC/D3 cells, resulting in increased radiolabelled sucrose concentration and decreased protein values in the wells (see Fig 7).

Further, the use of specific OCT isoform inhibitors, N-methylnicotinamide (OCT2) and corticosterone (OCT3), did not seem to cause any significant changes to the distribution of the drug pentamidine. However, treatment with amantadine (OCT1 and OCT2 inhibitor) significantly reduced pentamidine accumulation in both cell lines. Therefore we concluded that OCT1 is the key transporter present in both cell lines which was detected predominantly at the luminal membrane compared to the abluminal membrane in hCMEC/D3 cells by TEM. A previous study by Lin et al. (2010) [11] also found that OCT1 and OCT2 were mainly expressed on the luminal side of human brain microvessel endothelial cells and adult rat brain endothelial cells using confocal microscopy and Western blot analysis. This polarised expression of OCT1 would explain the accumulation of pentamidine observed *in vivo* in brain endothelial cells by Sanderson et al. (2009) [2].

There are several different transporters that are expressed at the BBB that can also transport positively charged molecules. Novel OCTs (OCTNS) have a high similarity to the amino acid sequence of OCT1 and OCT2 [44] and OCTN1 and 2 (SLC22A4 and A5) have been found to be expressed in the luminal membrane of brain capillary endothelial cells[45];[46]. OCTNs are involved in the sodium-dependent or independent transport of zwitterions and also function as an organic cation/proton exchanger. In addition, the substrates of MATEs are very similar to OCTs. [47] carried out mRNA expression profiles in brain microvessels extracted human brain cortex samples and detected the presence of MATE1 and MATE2 (SLC47A1 and A2). MATE1 expression was also confirmed by confocal image analysis. In contrast, [48] concluded that MATE1 was not expressed at the mouse BBB.

PMAT (SLC29A4) is another organic cation transporter that is sodium-independent, with low-affinity and high capacity for its substrates. It transports typical organic cations such as MPP+ and TEA making it functionally very similar to OCTs [49]. PMAT mRNA has also been identified in brain microvessels, brain microvascular endothelial cells, astrocytes, and pericytes isolated from C57BL/6 mice and Wistar rats. Western blot analysis localised PMAT to the luminal and abluminal sides of the mouse, rat, and human BBB. PMAT’s transport of organic cations was observed to be enhanced in acidic pH and therefore PMAT may be proton-dependent when transporting its substrates [50][51]. Thus, the substrates could be effluxed from the brain endothelial cells since the intracellular pH of the brain endothelial cells was found to be lower than the extracellular pH [52]. Our functional *in vitro* studies in both the hCMEC/D3 and bEnd.3 cells revealed the absence of an effect of OCTN1, OCTN2, MATE1, MATE2 and PMAT inhibitors on [^3^H]pentamidine accumulation.

The involvement of other uptake transporters for pentamidine at the mammalian BBB was also investigated. Since adenosine-sensitive transporters are implicated in pentamidine transport into the parasite *T*.*b*.*brucei*, the adenosine sensitivity of pentamidine transport at the human BBB was also tested to determine if it uses the ENT (SLC29A1, A2) or CNT (SLC28A2) system that transports nucleosides and nucleoside-derived drugs. However, no significant effects on [^3^H]pentamidine accumulation were observed under these treatment conditions. This could indicate that adenosine sensitive transporters are not involved in pentamidine accumulation in the brain endothelial cells, albeit at the BBB there is evidence for the presence of ENT1, ENT2 and CNT2 at both transcript and protein levels as found in primary culture of rat brain endothelial cells[53].

The data shown in this study highlights crucial differences between two *in vitro* models of the BBB–human and mouse. Species and/or culturing differences may lead to differences in the expression of transporters that the current study highlights in the case of ABC transporters. It is therefore important to understand the limitations of each model and employing several models and techniques to understand drug transport, as is the case in this study, may help reach a firmer conclusion.

The results here suggest that pentamidine is a substrate for OCT1 transporter at the BBB and is then effluxed by ATP-dependent mechanisms, most likely P-gp. It is interesting that pentamidine transport into the endothelial cells is very effective but it does not translate to therapeutic concentrations inside the brain. We suggest two reasons for this phenomenon: a) there is a small component of ATP-dependent efflux occurring at the luminal membrane which extrudes a proportion of pentamidine molecules back into the blood and b) the presence of OCT1, the main uptake transporter for pentamidine, has been shown to be predominantly at the luminal membrane in hCMEC/D3 in this study as well as in brain microvessel endothelial cells obtained from humans, mice, and rats as shown by Lin et al. (2010) [11], which results in increased uptake at the luminal membrane but not at the abluminal membrane. Together, the result is the accumulation of pentamidine in brain microvascular endothelial cells and subsequent return to the blood, and little movement into the brain across the abluminal membrane. This is probably why pentamidine is very effective in treating early-late stage trypanosomiasis. In addition, the interaction of pentamidine with OCT1 at the membrane makes OCT1 a potential target to reduce pentamidine interaction with the pancreas beta cells that often leads to diabetes mellitus, one of pentamidine’s side-effects [54][55]. Nonetheless, the true burden of HAT is poorly reflected in many existing assessments [56]. New, improved and safer HAT treatments are urgently required. This study provides information that is of interest to those developing new diamidine analogues plus those who are interested in repurposing pentamidine for other disease paradigms.

## Supporting information

S1 File **Fig A**. Confluent monolayer of hCMEC/D3 cells growing on a transwell polycarbonate filter as seen by transmission electron microscopy. The tight junction has been marked with an arrow. Magnification– 3500x. **Fig B.** Confluent monolayer of bEnd.3 cells growing on a transwell polycarbonate filter as seen by transmission electron microscopy. The tight junction has been marked with an arrow. Magnification– 3500x. **Fig C**. Effect of CNT inhibitors and a polyamine substrate on [^3^H] pentamidine accumulation in both cell lines.100 μM adenosine (CNT and ENT substrate) and 50 μM fludarabine (CNT 1 and 2 inhibitor) were used with [^3^H]pentamidine and [^14^C]sucrose and was found not to affect pentamidine accumulation in both cell lines. All data expressed as mean ± S.E.M, n = 3–4 passages of cells, with 6 replicates (wells) per timepoint per plate. Data were analysed with SigmaPlot 13.0. **Table A.** Primary and secondary antibodies used for protein expression studies. The antibodies for WB were all made up in PBS-T with 5% BSA and for IF in PBS+ with 5% goat serum. **Table B**. Accumulation buffer composition (pH ~ 7.45). **Table C.** Artificial plasma composition (pH ~ 7.45). The artificial plasma consisted of a modified Krebs-Henseleit mammalian Ringer solution with the following constituents dissolved in distilled water. **Table D.** Physiological buffer (capillary depletion buffer) (pH ~ 7.45) constituents were dissolved in distilled water.
(DOCX)

## Abbreviations

2-DG: 2-deoxy-d-glucose
ABC: ATP-Binding Cassette
AQP2: Aquaglyceroporin 2
BBB: Blood-Brain Barrier
BCRP: Breast-cancer Resistance Protein
CNT: Concentrated Nucleoside Transporter
DMEM: Dulbecco’s Modified Eagle Medium
DMSO: Dimethyl Sulfoxide
ENT: Equilibrative Nucleoside Transporter
HAPT1: High Affinity Pentamidine Transporter 1
HRP: Horseradish Peroxidase
IF: Immunofluorescence
LAPT1: Low Affinity Pentamidine Transporter 1
MATE: Multidrug and Toxin Extrusion Transporters
MRP: Multidrug Resistance-associated Protein
MTT: (3-(4,5-Dimethylthiazol-2-yl)-2,5-diphenyltetrazolium bromide
OCT: Organic Cation Transporter
OCTN: Organic Cation Transporter Novel
P-gp: P-glycoprotein
PMAT: Plasma membrane monoamine transporter
PVDF: Polyvinylidene difluoride
RIPA: Radioimmunoprecipitation assay buffer
SLC: Solute-carrier
T.b: *Trypanosoma brucei*
TEM: Transmission Electron Microscopy
V_d_: Volume of Distribution
WB: Western Blot
WGA: Wheat-germ Agglutinin
