# Supplementary material for: Organic cation transporter 1 (OCT1) is involved in pentamidine transport at the human and mouse blood-brain barrier (BBB)
Source: PLoS One. 2017 Mar 31;12(3):e0173474. doi: 10.1371/journal.pone.0173474 (PMC5376088; doi:10.1371/journal.pone.0173474)
Supplement: S1 File — Fig A. Confluent monolayer of hCMEC/D3 cells growing on a transwell polycarbonate filter as seen by transmission electron microscopy. The tight junction has been marked with an arrow. Magnification– 3500x. Fig B. Confluent monolayer of bEnd.3 cells growing on a transwell polycarbonate filter as seen by transmission electron microscopy. The tight junction has been marked with an arrow. Magnification– 3500x. Fig C. Effect of CNT inhibitors and a polyamine substrate on [3H] pentamidine accumulation in both cell lines.100 μM adenosine (CNT and ENT substrate) and 50 μM fludarabine (CNT 1 and 2 inhibitor) were used with [3H]pentamidine and [14C]sucrose and was found not to affect pentamidine accumulation in both cell lines. All data expressed as mean ± S.E.M, n = 3–4 passages of cells, with 6 replicates (wells) per timepoint per plate. Data were analysed with SigmaPlot 13.0. Table A. Primary and secondary antibodies used for protein expression studies. The antibodies for WB were all made up in PBS-T with 5% BSA and for IF in PBS+ with 5% goat serum. Table B. Accumulation buffer composition (pH ~ 7.45). Table C. Artificial plasma composition (pH ~ 7.45). The artificial plasma consisted of a modified Krebs-Henseleit mammalian Ringer solution with the following constituents dissolved in distilled water. Table D. Physiological buffer (capillary depletion buffer) (pH ~ 7.45) constituents were dissolved in distilled water. (DOCX) [file pone.0173474.s001.docx]

**Organic Cation Transporter 1 (OCT1) involved in pentamidine transport at the human and mouse blood-brain barrier (BBB)**

**Authors:** Gayathri N. Sekhar^1^, Lisa Sanderson^1^, Gema Vizcay-Barrena^2^, Ana R. Georgian^1^, Rachel C. Brown^1^, Paula Murersan^1^, Roland A. Fleck^2^, Sarah A. Thomas^1^

^1^*Institute of Pharmaceutical Science, King’s College London, Waterloo, London, SE1 9NH UK*

*^2^Centre for Ultrastructural Imaging, King’s College London, London Bridge, SE1 1UL UK*

**Table A** Primary and Secondary antibodies used for protein expression studies. The antibodies for WB were all made up in PBS-T with 5% BSA and for IF in PBS+ with 5% goat serum.

| PROTEIN | Primary Antibody | Secondary Antibody WB | Secondary antibody IF |
| --- | --- | --- | --- |
| P-gp | Rabbit monoclonal anti-human and mouse (Abcam, ab170904)  WB Dilution - 1:2000  IF Dilution - 1:200 | Goat anti-rabbit HRP (Abcam, ab6721) Dilution - 1:3000 | Goat anti-rabbit Alexa Fluor^®^ 488 (Abcam, ab181448)  Dilution – 1:200 |
| BCRP | Rabbit polyclonal anti-human and mouse (New England Biolabs, 4477S) –  WB dilution - 1:2000  IF dilution – 1:200 | Goat anti-rabbit HRP (Abcam, ab6721) Dilution- 1:2000 | Goat anti-rabbit Alexa Fluor^®^ 488 (Abcam, ab181448)  Dilution – 1:200 |
| MRP4 | Rat monoclonal [M4I-10] anti-human and mouse (Abcam 15602)  IF dilution - 1:200 |  | Rabbit anti-rat Alexa Fluor^®^ 488 (Abcam, ab6734)  Dilution – 1:200 |
| OCT-1 (SLC22A1) | Rabbit polyclonal anti-human and mouse (Abcam, ab55916)  WB Dilution- 1:250 | Goat anti-rabbit HRP (Abcam, ab6721) Dilution - 1:1000 |  |
| OCT-2 (SLC22A2) | Rabbit monoclonal to human and mouse (Abcam, ab170871)  WB Dilution – 1:2000 | Goat anti-rabbit HRP (Abcam, ab6721) Dilution - 1:2000 |  |
| OCT -3 (SLC22A3) | Rabbit polyclonal to human and mouse (Abcam, ab183071), WB Dilution – 1:600 | Goat anti-rabbit HRP (Abcam, ab6721) Dilution - 1:2000 |  |
| α-tubulin | Rabbit monoclonal to human and mouse (Millipore Limited, UK)  WB Dilution - 1:10000 | Goat anti-rabbit HRP (Abcam, ab6721) Dilution - 1:10000 |  |
| GAPDH | Rabbit polyclonal to GAPDH (Abcam, ab9485)  WB Dilution 1: 2500 |  |  |
| Wheat Germ Agglutinin (WGA) |  |  | Tetramethylrhodamine conjugated (Life Technologies, Paisley, UK)- 1:200 |

**Table B** Accumulation buffer composition (pH ~ 7.45).

| **Concentration** | **Constituents dissolved in distilled water** |
| --- | --- |
| 135mM | NaCl |
| 10mM | HEPES |
| 5.4mM | KCl |
| 1.5mM | CaCl_2_ |
| 1.2mM | MgCl_2_ |
| 1.1mM | D-glucose |

**Table C** Artificial plasma composition (pH ~ 7.45).

The artificial plasma consisted of a modified Krebs-Henseleit mammalian Ringer solution with the following constituents dissolved in distilled water.

| **Concentration** | **Constitutents dissolved in distilled water** |
| --- | --- |
| 117 mM | NaCl |
| 4.7 mM | KCl |
| 2.5 mM | CaCl_2_ |
| 1.2 mM | MgSO_4_ |
| 24.8 mM | NaHCO_3_ |
| 1.2 mM | KH_2_PO_4_ |
| 10 mM | glucose |
| 1 g/liter | bovine serum albumin |

**Table D** Physiological buffer (capillary depletion buffer) (pH ~ 7.45) constituents were dissolved in distilled water.

| **Concentration** | **Constituents dissolved in distilled water** |
| --- | --- |
| 141 mM | NaCl |
| 4 mM | KCl |
| 1 mM | MgSO_4_.7H_2_O |
| 10.9 mM | HEPES |
| 1mM | NaH_2_PO_4_.2H_2_O |
| 2.8mM | CaCl_2_ |
| 10mM | glucose |

**
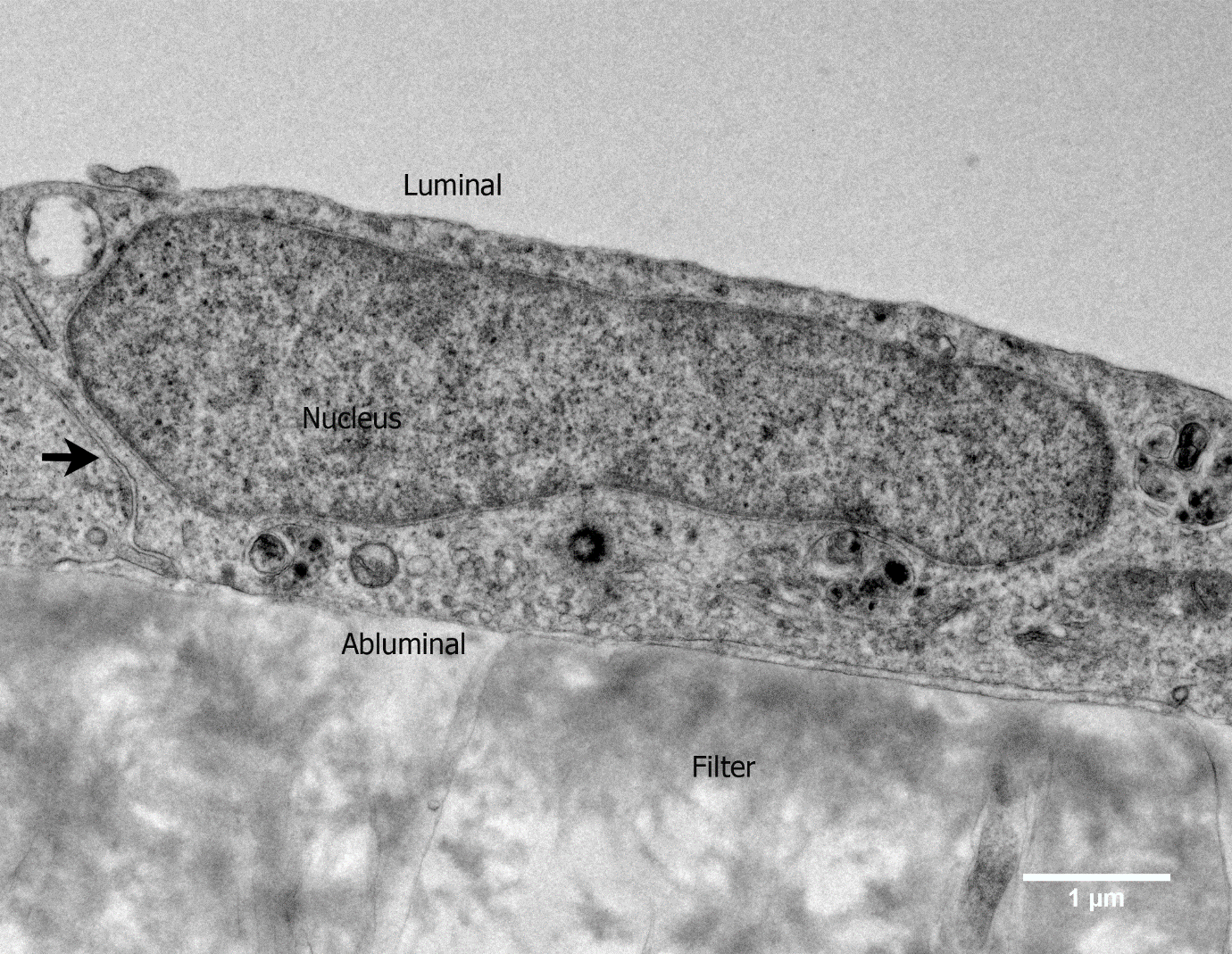
**

**Fig A** Confluent monolayer of hCMEC/D3 cells growing on a transwell polycarbonate filter as seen by transmission electron microscopy. The tight junction has been marked with an arrow. Magnification – 3500x.


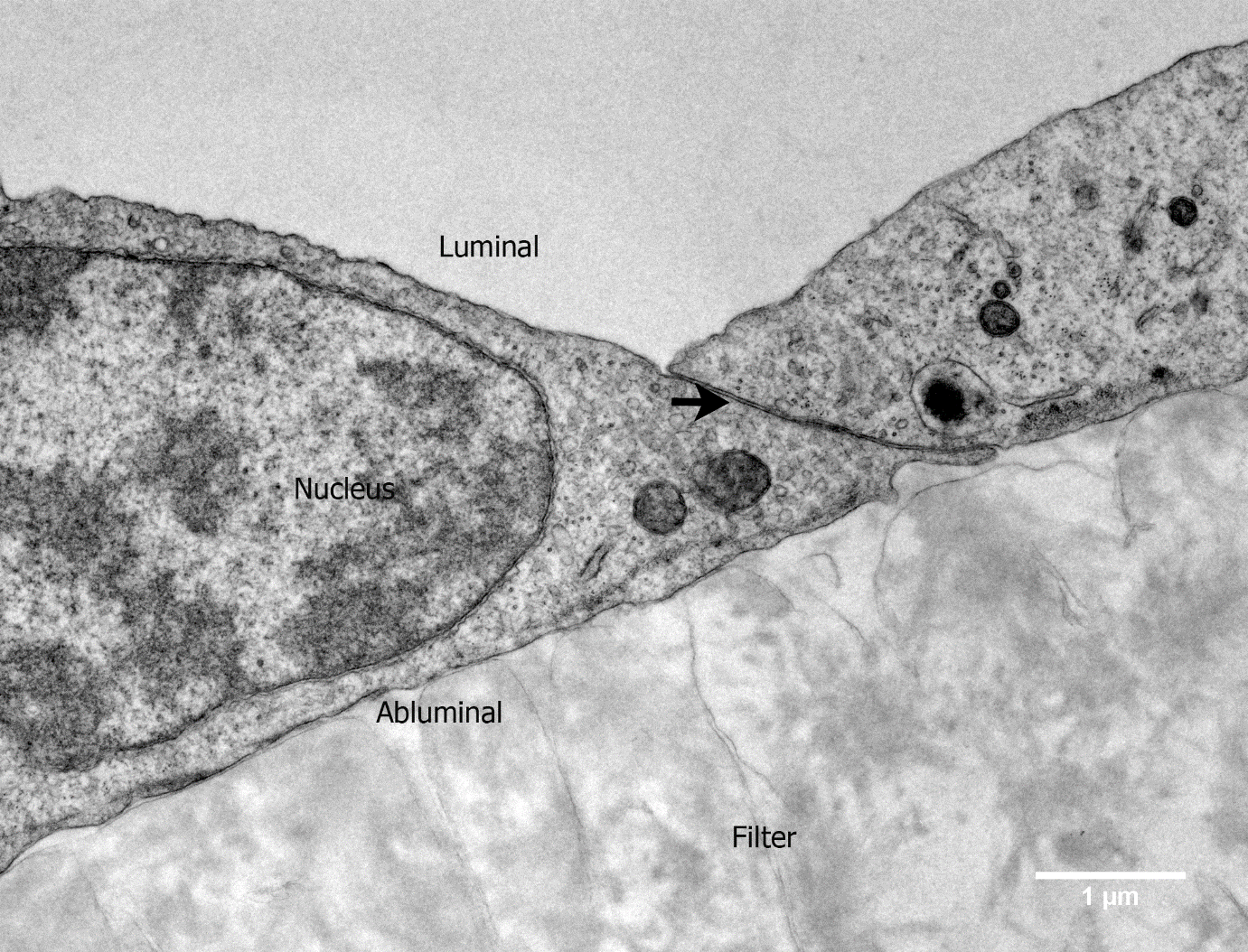


**Fig B** – Confluent monolayer of bEnd.3 cells growing on a transwell polycarbonate filter as seen by transmission electron microscopy. The tight junction has been marked with an arrow. Magnification – 3500x.





**Fig C -** Effect of CNT inhibitors and a polyamine substrate on [^3^H] pentamidine accumulation in both cell lines.100 µM adenosine (CNT and ENT substrate) and 50 µM fludarabine (CNT 1 and 2 inhibitor) were used with [^3^H]pentamidine and [^14^C]sucrose and was found not to affect pentamidine accumulation in both cell lines. All data expressed as mean ± S.E.M, n =3-4 passages of cells, with 6 replicates (wells) per timepoint per plate. Data were analysed with SigmaPlot 13.0.
